#  Pharmacovigilance Analysis of Serious Adverse Events Reported for Biologic Response Modifiers Used as Prophylaxis against Transplant Rejection: a Real-World Postmarketing Experience from the US FDA Adverse Event Reporting System (FAERS) 

**Published:** 2013-05-01

**Authors:** A. K. Ali

**Affiliations:** Department of Pharmaceutical Outcomes & Policy, College of Pharmacy, University of Florida, USA

**Keywords:** Immunosuppressive medications, Biologic response modifiers, Pharmacovigilance, Serious adverse events, FAERS

## Abstract

Background: Immunosuppression by biologic response modifiers (BRM) is a crucial component for successful organ transplantation. In addition to their variable effectiveness in the prevention of organ rejection, these medications have safety concerns that complicate therapeutic outcomes in organ transplant patients.

Objective: This study aims at identifying and characterizing safety signals of serious adverse events associated with exposure to BRM among organ transplant patients in a real-world environment.

Methods: The FDA Adverse Event Reporting System was utilized to apply a pharmacovigilance disproportionality analysis to indentify serious adverse events. Associations between drugs and events were measured by empirical Bayes geometric mean (EBGM) and the corresponding 95% confidence intervals (EB05–EB95). Associations with EBGM≥2 were considered significant safety signals.

Results: From 1997 to 2012, a total of 12,151 serious adverse event reports for BRM were reported; 15.6% of them (n=1,711) met the safety signal threshold of EB05>1, and 11.6% of these signals (n=199) were significant (EBGM≥2). Sirolimus and mycophenolate accounted for the majority of all signals; antithymocyte immunoglobulin (ATI) and cyclosporine contributed to the majority of significant signals. The following significant signals were identified for ATI (reduced therapeutic response, pulmonary edema, hypotension, serum sickness, infusion-related reaction, and anaphylactic reaction); for azathioprine (alternaria infection, fungal skin infection, and lymphoproliferative disorder); for cyclosporine (neurotoxicity, graft vs. host disease, and thyroid cancer); for cyclophosphamide (disease progression); for daclizumab (cytomegalovirus infection); and for tacrolimus (coma and tremor). 33.6% of these events contributed to patient death (n=67); 6.5% were life-threatening (n=13); 32.1% lead to hospitalization (n=64); and 27.6% resulted in other serious outcomes (n=55).

Conclusion: Utilization of BRM for the prophylaxis against transplant rejection is associated with serious adverse events that could be fatal.

## INTRODUCTION

End-stage organ failure is a common problem with limited treatment approaches beyond organ transplantation [1]. Number of candidates on waiting lists for transplantation continues to rise, while number of donors continues to level off. In the United States, there were 11,663 organ donors and 23,360 organ transplants from January to October 2012 [2]. As of January 2013, there were 116,944 candidates on waiting lists with 74,451 (63.6%) being classified as active waitlisted who were eligible for organ offer at a given point of time [2]. In 2007, approximately 2.5 million individuals with end-stage organ failure died [3]; nevertheless, pre-transplantation mortality rates were reduced among patients on waiting lists across all solid organs [4]. From 2010 to 2011, the number of patients on the waiting list for organ transplantation in the United States increased by 0.2% from 54,505 to 54,599; but the number of organ transplantations declined by 0.7% from 17,726 to 17,604 [4]. Organ transplantation improved patient’s quality of life and overall survival; however, organ rejection by the host’s immune system is a major complication of organ transplantation [1, 4]. Among adult transplant patients, the approximate incidence rates of acute rejection within the first year of transplantation are 40% for intestine, 19% for heart, 18% for lung, 15%–20% for pancreas, 15% for liver, and 10% for kidney [4].

Immunosuppressive therapy aims to provide minimum suppression to the immune system to prevent transplant rejection while avoiding or minimizing complications of immunodeficiency. Generally, immunosuppressive medications are classified into corticosteroids (*e.g.*, prednisolone) and biologic response modifiers (BRM) (*e.g.*, cyclosporine). The introduction of BRM as an alternative to corticosteroids with its associated metabolic adverse reactions, is considered a breakthrough in prophylaxis against transplant rejection. However, these agents are associated with a myriad of safety concerns and not free from serious adverse outcomes that could complicate transplantation [5]. Some adverse reactions are well recognized for these agents; nonetheless, serious events are not well documented. By utilizing real-world data, this study aims to identify and characterize significant safety signals of serious adverse events reported for BRM used for the prophylaxis against transplant rejection.

## Materials and Methods

Unduplicated adverse event reports spontaneously submitted to the United States Food and Drug Administration (FDA) Adverse Event Reporting System (FAERS) (formerly AERS) from October 1, 1997 to March 31, 2012 were used to apply a pharmacovigilance disproportionality analysis for the detection and characterization of serious adverse events associated with biologic response modifiers indicated for the prophylaxis against organ rejection. The FAERS is a database of spontaneously submitted adverse event reports for pharmaceutical products that is updated on a quarterly basis by FDA. Reports are submitted from health care professionals, consumers or caregivers, manufacturers, and other sources from the United States and other countries [6]. The FAERS is considered the primary source for the FDA to manage and monitor new adverse events reported for marketed pharmaceutical products [7].

**Identification of biologics response modifiers**

**Figure 1:**
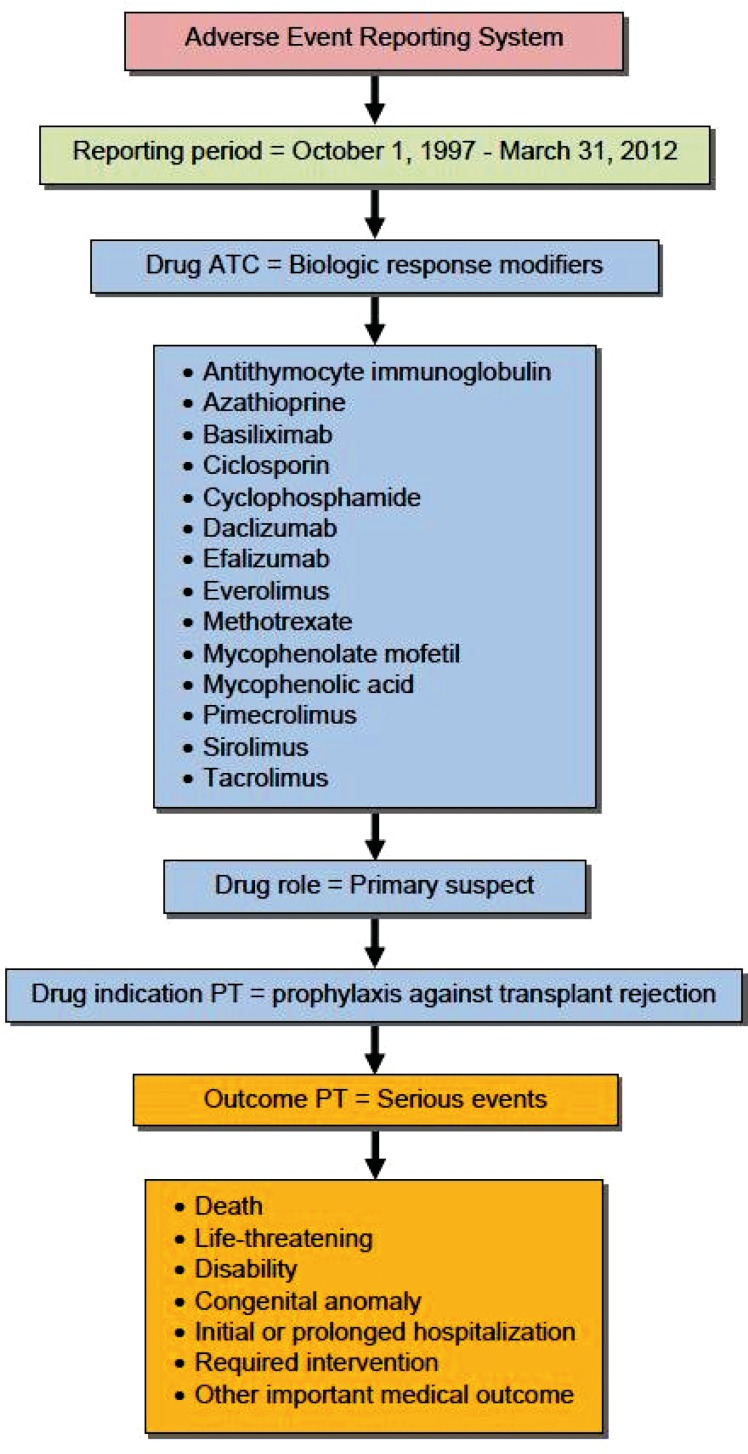
Database restriction criteria

The World Health Organization’s Anatomical Therapeutic Chemical (ATC, January 2012) classification system was used to identify BRM. Table 1 lists individual agents approved for marketing in the United States. Adverse event reports that included BRM as primary suspects in the occurrence of the adverse event, and those with an indication for the prophylaxis against transplant rejection were included in the analysis. Figure 1 shows the applied database restriction criteria.

**Table 1:** Biologic response modifiers currently available in the United States (Source: www.fda.gov).

Class	Agent	Brand name example*	FDA approval date
Alkylating agents	Cyclophosphamide	Cytoxan	Nov. 1959
Antimetabolites	Azathioprine	Imuran	Mar. 1968
	Methotrexate	Mexate	Sept. 1979
Calcineurin inhibitors	Cyclosporine	Sandimmune	Nov. 1983
	Tacrolimus	Prograf	Apr. 1994
IL-2R antibodies	Basiliximab	Simulect	May 1998
	Daclizumab	Zenapax	Dec. 1997
	Efalizumab	Raptiva	Oct. 2003
mTOR inhibitors	Everolimus	Afinitor	Mar. 2009
	Pimecrolimus	Elidel	Dec. 2001
	Sirolimus	Rapamune	Sept. 1999
	Temsirolimus	Torisel	May 2007
Purine synthesis inhibitors	Mycophenolic acid	Myfortic	Feb. 2004
	Mycophenolate mofetil	Cellcept	May 1995
T-cell depletion antibodies	Antithymocyte immunoglobulin	Thymoglobulin	Nov. 1981

FDA: Food and Drug Administration.

IL-2R: Interleukin-2 Receptor.

mTOR: Mammalian Target of Rapamycin.

* Product brand names are the property of their respective manufacturers.

**Identification of serious adverse events**

The Preferred Term (PT) hierarchy of the Medical Dictionary for Regulatory Activities (MedDRA 15.0, March 2012) was used to identify serious adverse events that resulted in death, life-threatening experience, persistent or significant disability or incapacity, congenital anomaly or birth defect, initial or prolonged existing inpatient hospitalization, requirement for intervention, or any other important medical outcomes [8]. Within these reports, safety signals were evaluated for specific adverse events, and event PTs with significant safety signals were discussed in this report. Within FAERS, adverse events, seriousness outcome, and clinical indication for the reported drug were recorded using the PT hierarchy of MedDRA [6].

**Pharmacovigilance disproportionality analysis**

Empirica Signal (7.3.341, November 2011, Oracle USA, Inc., Redwood City, CA, USA) was used to generate empirical Bayesian geometric mean (EBGM) and its corresponding 95% confidence intervals (EB05–EB95). An EBGM >1 was interpreted as “the reported adverse event for the corresponding drug was higher than that expected compared to other drugs and events in the database.” Safety signals were identified if the lower limit of the 95% confidence interval (EB05) was >1, and drug-event combinations with EBGM ≥2 were considered significant safety signals [9, 10]. Signals with both EBGM and EB05 values ≥2 were considered significant safety signals that warrant regulatory action [6].

## RESULTS

**Overview of serious adverse events reported for BRM**

During the study period, a total of 12,151 adverse event reports was submitted for BRM and were classified as serious events. More than half of these serious events were attributed to sirolimus (n=6,749); about 19% were for mycophenolic acid (n=2,317); and about 9%, 7%, and 6% of serious event reports were respectively, for cyclosporine (n=1,067), tacrolimus (n=841), and antithymocyte immunoglobulin (n=725). The rest of BRM collectively contributed to 4% of the reports (n=452) (Table 2).

**Table 2:** Distribution of serious adverse event reports for biologic response modifiers

**Drug**	**Number of Serious Event Reports (%)**		
	**All Serious Events** **(EB05 >0)** **n=12,151**	**All Signals** **(EB05 >1)** **n=1,711**	**Significant Signals** **(EBGM ** **≥** **2)** **n=199**
Antithymocyte immunoglobulin	725 (5.9)	172 (10.0)	68 (34.1)
Azathioprine	99 (0.8)	36 (2.1)	18 (9.0)
Cyclosporine	1,067 (8.8)	113 (6.6)	58 (29.1)
Cyclophosphamide	60 (0.5)	9 (0.5)	9 (4.5)
Daclizumab	267 (2.2)	22 (1.3)	22 (11.0)
Everolimus	5 (0.04)	0 (0.0)	0 (0.0)
Mycophenolic Acid	2,317 (19.0)	304 (18.0)	0 (0.0)
Sirolimus	6,749 (55.0)	896 (52.0)	0 (0.0)
Tacrolimus	841 (6.9)	159 (9.3)	24 (12.0)
Tacrolimus and Sirolimus	21 (0.2)	0 (0.0)	0 (0.0)

EBGM: Empirical Bayes geometric mean

EB05: Lower limit of 95% confidence interval

n: Number of reports within the corresponding category

**Safety signals of serious adverse events for BRM (EB05>1)**

**Figure 2:**
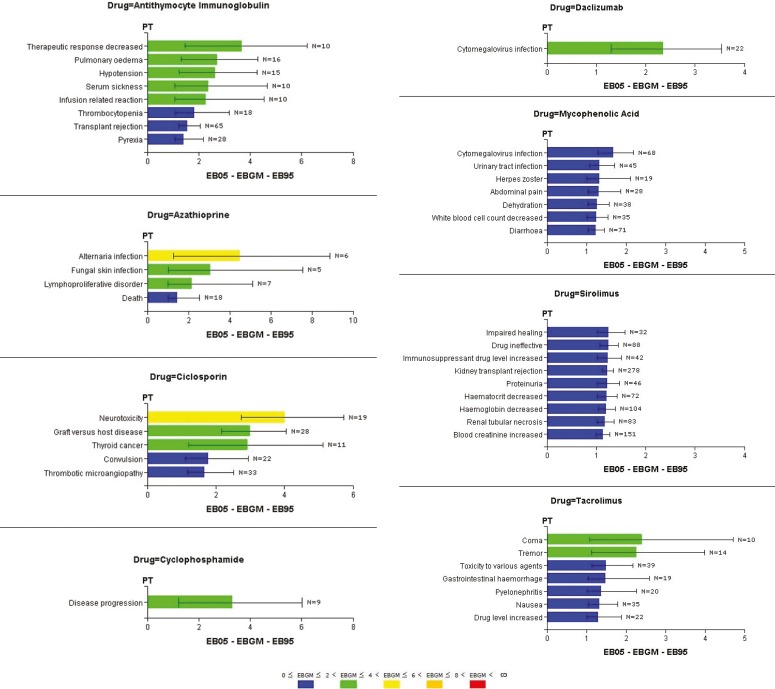
Signals of serious adverse events associated with biologic response modifiers

Among the identified serious adverse events, only 14% of the reports (n=1,711) generated safety signals of serious events associated with BRM with EB05 values >1. None of the reports indicated patient recovery from the serious events. Sirolimus and mycophenolic acid, respectively, contributed to 52% (n=896) and 18% (n=304) of the signals. Antithymocyte immunoglobulin, tacrolimus, and cyclosporine accounted for 10% (n=172), 9% (n=159), and 7% (n=113) of the signals, respectively; the rest of the BRM collectively contributed to 4% (n=67) of the identified signals (Table 2). Figure 2 shows safety signals with EB05 values >1 for reported serious events associated with BRM. For antithymocyte immunoglobulin, eight signals were identified—the strongest signal was for a MedDRA PT “therapeutic response decreased,” which could be a proxy for immunosuppression failure (EBGM=3.67; EB05–EB95: 1.47–6.22). Azathioprine was associated with four signals with the strongest signal for “alternaria infection” (EBGM=4.50; EB05–EB95: 1.27–8.88). Cyclosporine was associated with five signals and “neurotoxicity” was the strongest identified signal (EBGM=4.02; EB05–EB95: 2.74–4.74). Cyclophosphamide and daclizumab each associated with one safety signal; a signal of “disease progression” was identified for cyclophosphamide, which can also be a proxy for immunosuppression failure (EBGM=3.31; EB05–EB95: 1.22–6.03), and a signal of “cytomegalovirus infection” was identified for daclizumab (EBGM=2.36; EB05–EB95: 1.30–3.53). Among the seven signals identified for mycophenolic acid, “cytomegalovirus infection” was the strongest (EBGM=1.69; EB05–EB95: 1.30–2.19). Sirolimus was associated with nine signals, which were close in values; however, “impaired healing” (EBGM=1.26; EB05–EB95: 1.01–1.59) and “drug ineffective” (EBGM=1.25; EB05–EB95: 1.07–1.44) were the strongest. Tacrolimus was associated with seven safety signals for serious events; the strongest signal was for “coma” (EBGM=2.40; EB05–EB95: 1.08–4.71). Conversely, safety signals were generated neither for everolimus (EBGM=1.22; EB05–EB95: 0.88–2.40) nor for concomitantly administered sirolimus and tacrolimus (EBGM=1.16; EB05–EB95: 0.85–2.04).

**Significant safety signals of serious adverse events for BRM (EBGM** **>** **2)**

About 12% of the identified signals were significant (n=199) with a total number of 16 significant signals for six BRM (Tables 2 and 3). The distribution of significant signals in relation to the identified signals for individual BRM was as follows: about 40% of signals for antithymocyte immunoglobulin (n=68), half the signals for azathioprine (n=18) and cyclosporine (n=58), all the signals for cyclophosphamide (n=9) and daclizumab (n=22), and 15% of the signals for tacrolimus (Table 2). Although mycophenolic acid and sirolimus showed safety signals of serious adverse events, none of these signals were significant.

**Table 3:** Significant safety signals of serious adverse events associated with biologic response modifiers (EBGM ≥2)

**Drug**	**Adverse event PT**	**No. of reports** *	**EBGM (EB05–EB95)**
Antithymocyte immunoglobulin	Therapeutic response decreased	10	3.67 (1.47–6.22)
	Pulmonary edema	16	2.73 (1.31–4.28)
	Hypotension	15	2.64 (1.25–4.28)
	Serum sickness	10	2.37 (1.08–4.67)
	Infusion related reaction	10	2.26 (1.07–4.54)
	Anaphylactic reaction	7	2.10 (1.00–5.01)
Azathioprine	Alternaria infection	6	4.50 (1.27–8.88)
	Fungal skin infection	5	3.06 (1.03–7.55)
	Lymphoproliferative disorder	7	2.17 (1.00–5.14)
Cyclosporine	Neurotoxicity	19	4.02 (2.74–5.74)**
	Graft versus host disease	28	3.00 (2.18–4.06)**
	Thyroid cancer	11	2.92 (1.20–5.12)
Cyclophosphamide	Disease progression	9	3.31 (1.22–6.03)
Daclizumab	Cytomegalovirus infection	22	2.36 (1.30–3.53)
Tacrolimus	Coma	10	2.40 (1.08–4.71)
	Tremor	14	2.27 (1.13–3.99)

EBGM: Empirical Bayes geometric mean

EB05–EB95: 95% confidence interval

PT: Preferred term

* Total number of reports with significant signals (n=199)

** Significant safety signals that warrant regulatory action (both EBGM and EB05 ≥2)

Table 3 lists the identified significant signals for individual BRM by the decreasing order of signal strength within each agent. The reporting of the following adverse events was significantly higher than that expected for the following individual BRM: for antithymocyte immunoglobulin, “therapeutic response decreased” (EBGM=3.67; EB05–EB95: 1.47–6.22), “pulmonary edema” (EBGM=2.73; EB05–EB95: 1.31–4.28), “hypotension” (EBGM=2.64; EB05–EB95: 1.25–4.28), “serum sickness” (EBGM=2.37; EB05–EB95: 1.08–4.67), “infusion related reaction” (EBGM=2.26; EB05–EB95: 1.07–4.54), and “anaphylactic reaction” (EBGM=2.10; EB05–EB95: 1.07–5.01); for azathioprine, “alternaria infection” (EBGM=4.50; EB05–EB95: 1.27–8.88), “fungal skin infection” (EBGM=3.06; EB05–EB95: 1.03–7.55), and “lymphoproliferative disorder” (EBGM=2.17; EB05–EB95: 1.00–5.14); for cyclosporine, “neurotoxicity” (EBGM=4.02; EB05–EB95: 2.74–5.74), “graft *vs*. host disease” (EBGM=3.00; EB05–EB95: 2.18–4.06), and “thyroid cancer” (EBGM=2.92; EB05–EB95: 1.20–5.12); and for tacrolimus, “coma” (EBGM=2.40; EB05–EB95: 1.08–4.71), and “tremor” (EBGM=2.27; EB05–EB95: 1.13–3.99). All the identified signals of “disease progression” for cyclophosphamide and “cytomegalovirus infection” for daclizumab were significant. Furthermore, significant signals that necessitate regulatory follow-up were only identified for cyclosporine in association with “neurotoxicity” and “graft versus host disease” adverse events (both EBGM and EB05 values exceeded 2).

Table 4 shows the characteristics of BRM adverse event reports in which significant signals were identified. Approximately 34% of these events contributed to patient death (n=67); 6.5% were life-threatening (n=13); 32.1% led to hospitalization or required interventions (n=64); and 27.6% contributed to other serious outcomes (n=55). None of the events resulted in disabilities or congenital anomalies. About 46% of death reports were attributed to cyclosporine (n=31), 15% to antithymocyte immunoglobulin (n=10), 13.4% to cyclophosphamide (n=9), 12% to tacrolimus (n=8), 9% to azathioprine (n=6), and 4.4% to daclizumab (n=3). The vast majority of the reported life-threatening events were for antithymocyte immunoglobulin (n=12); only one report was for tacrolimus. About 42% of the reported hospitalizations or intervention requirements were attributed to antithymocyte immunoglobulin (n=27), 29.6% to daclizumab (n=19), 17.1% to tacrolimus (n=11), 7.8% to cyclosporine (n=5), and 3.1% to azathioprine (n=2).

The median age for patients exposed to antithymocyte and experienced serious events that generated significant safety signals was 47.5 years; it was 64 years for azathioprine users, 37 for cyclosporine users, 48 for daclizumab users, and 54 years for tacrolimus users. About 54% of patients in antithymocyte immunoglobulin reports were males (n=37), 40% were females (n=27), and 6% with unknown sex (n=4). About 78% of reported azathioprine users were males (n=14), 17% were females (n=3), and one patient with unknown sex. Approximately 45% of the reported cyclosporine users were males (n=26), 7% were females (n=4), and 48% with unknown sex (n=28). Among reports for daclizumab, about 73% of patients were males (n=16), 18% were females (n=4), and 9% with unknown sex (n=2). Half of the patients in tacrolimus reports were males (n=12), 37.5% were females (n=9), and 12.5% had unknown sex reported (n=3). Patient demographics were not reported for cyclophosphamide users.

The median number of medications concurrently administered with the respective BRM was three for reports of antithymocyte immunoglobulin, two for azathioprine and cyclophosphamide, one for cyclosporine, nine for daclizumab, and six for tacrolimus. Over 17% of antithymocyte immunoglobulin serious reports with significant signals did not have concomitantly used medications (n=12), corresponding to almost 57% of “anaphylactic reaction,” 40% of “infusion related reaction,” and 13% of each of “pulmonary edema” and “hypotension,” events. About 8% of tacrolimus reports did not have additional medications reported (n=2), corresponding to 10% of “coma,” and 7.1% of “tremor” events. Characteristics of individual adverse events for corresponding BRM are described in Table 4.

**Table 4:** Characteristics of serious adverse events for biologic response modifiers with significant safety signals (EBGM ≥2).

Drug	Adverse event (n)	Characteristics of reports							
		Seriousness, n (%)			Patient’s demographics			Co-drugs	
		Death	LT	HI	Age, year*	Sex**		No. of Co-drugs*	No. of reports without
						M	F		
Antithymocyte Ig	Therap. response decreased (10)	—	—	6 (60)	43 (17–57)	5 (50)	5 (50)	2 (2–4)	—
	Pulmonary edema (16)	4 (25)	2 (13)	6 (38)	54 (13–70)	7 (44)	9 (56)	7 (0–44)	2
	Hypotension (15)	2 (13)	6 (40)	5 (33)	52 (13–70)	11 (73)	3 (20)	4 (0–21)	2
	Serum sickness (10)	—	—	7 (70)	37 (24–61)	6 (60)	4 (40)	6 (3–38)	—
	Infusion related reaction (10)	2 (20)	1 (10)	1 (10)	47 (24–67)	4 (40)	3 (30)	2 (0–16)	4
	Anaphylactic reaction (7)	2 (29)	3 (43)	2 (29)	48 (15–52)	4 (57)	3 (43)	0 (0–20)	4
Azathioprine	Alternaria infection (6)	2 (33)	—	—	64 (28–67)	6 (100)	—	2 (2–3)	—
	Fungal skin infection (5)	2 (40)	—	—	65 (48–67)	5 (100)	—	2 (2–3)	—
	Lymphoproliferative disorder (7)	2 (29)	—	2 (39)	21 (5–37)	3 (43)	3 (43)	2 (1–7)	—
Cyclosporine	Neurotoxicity (19)	14 (74)	—	1 (5)	36 (17–49)	9 (47)	10 (53)	1 (1–4)	—
	Graft versus host disease (28)	17 (61)	—	4 (14)	37 (11–73)	12 (43)	12 (43)	1 (1–15)	—
	Thyroid cancer (11)	—	—	—	40 (23–57)	5 (45)	6 (55)	2	—
Cyclophosphamide	Disease progression (9)	9 (100)	—	—	U	U	U	2	—
Daclizumab	Cytomegalovirus infection (22)	3 (14)	—	19 (86)	48 (24–68)	16 (73)	4 (18)	9 (3–44)	—
Tacrolimus	Coma (10)	7 (70)	—	2 (20)	59 (46–75)	4 (40)	3 (30)	2 (0–12)	1
	Tremor (14)	1 (7)	1 (7)	9 (64)	49 (31–75)	8 (57)	6 (43)	10 (0–42)	1

F: Female

* Reported as median (minimum–maximum)

** Percentages out of total number of reports including unknown sex values.

## DISCUSSION

The United States FAERS database was used to conduct a retrospective pharmacovigilance analysis of serious adverse events reported for BRM immunosuppressive medications that are indicated for the prophylaxis against transplant rejection. The majority of identified significant safety signals contributed to patient death; however, these signals should not be interpreted as causal links between exposure to BRM and occurrence of serious adverse events. The identified adverse events were consistent with the known safety profile of individual BRM; however, the seriousness of these events, *e.g.*, death, is not established in the literature. For instance, proliferative disorders, *e.g.*, T-cell lymphoma [11], and opportunistic infections, *e.g.*, cytomegalovirus infection, are common complications of immunosuppression [12]. Also, transplant recipients are twice more likely to develop cancers than their counterparts without transplantation, and the risk increases greatly by infections with oncogenic viruses, *e.g.*, Epstein-Barr virus, Kaposi sarcoma herpes virus, and human papillomavirus [13]; this might be related to the duration and intensity of immunosuppression regardless of specific BRM [14], and therefore, the association of cyclosporine with thyroid cancer should be interpreted with caution. Although daclizumab was associated with significant signal of cytomegalovirus infection, clinical trials showed fewer incidences among daclizumab users compared to placebo users [15]. In general calcineurin inhibitors, *e.g.*, cyclosporine and tacrolimus, are associated with rare neurological and psychiatric adverse events, although coma and delirium have been reported for tacrolimus given at high doses [16]. Serums sickness and infusion-related reactions have been reported with antithymocyte immunoglobulin [17, 18], but the occurrence of anaphylactic reactions is not well documented.

Data repositories of spontaneously submitted adverse events, *e.g.*, FAERS are one of the key tools of routine assessment and management of risks associated with marketed pharmaceutical products. In addition to other limitations of spontaneously submitted adverse event data, these data are increasingly incomplete (*e.g.*, missing patient demographic information for cyclophosphamide), variable reporting rates overtime, underreporting, duplicate reports, unverified source of submitted data, inability to adjust for important confounders, and missing information about temporality [6]. Since data mining algorithms, *e.g.*, EBGM, are hypothesis generating techniques, they should not be used in isolation to clinical judgment and available epidemiological or clinical evidence. Furthermore, the estimated EBGM values should not be interpreted as incidence rates; rather they should be treated as the respective adverse event for the offending drug has been reported more than that expected compared to other adverse events and other drugs in the database during the specified reporting period. As an example of potential confounding effect by co-medications, systemic corticosteroids were mentioned in most of the reports with concurrent medications, and some reports included more than one class of BRM as secondary suspect in the occurrence of the adverse event.

In conclusion, utilization of BRM for the prophylaxis against transplant rejection is associated with serious adverse events that could be fatal and life-threatening. Transplant specialists should exercise caution when prescribing these medications to transplant patients and should monitor patient progress in terms of safety, tolerability and transplant outcomes throughout exposure period. Pharmacoepidemiological studies are required to evaluate the identified safety signals to help understand the benefit-risk profile of these medications.

## References

[1] Miller BW, Green GB, Harris IS, Lin GA, Moylan KC (2004). Solid Organ Transplant Medicine. The Washington Manual of Medical Therapeutics.

[2] Organ Procurement and Transplantation Network (OPTN). United Network for Organ Sharing (UNOS). Data as of January 30 2013. Rockville, MD: Department of Health and Human Services, Health Resources and Services Administration, Healthcare Systems Bureau, Division of Transplantation, 2013.

[3] US Government Information on Organ and Tissue Donation and Transplantation The Need is Real. Data. Rockville, MD: Department of Health and Human Services, Health Resources and Services Administration; 2012.

[4] Organ Procurement and Transplantation Network (OPTN) and Scientific Registry of Transplant Recipients (SRTR). OPTN/SRTR 2011 Annual Data Report. Rockville, MD: Department of Health and Human Services, Health Resources and Services Administration, Healthcare Systems Bureau, Division of Transplantation, 2012.

[5] National Guideline Clearinghouse (NGC) Guideline Summary: Guidelines on Renal Transplantation: Immunosuppression after Kidney Transplantation. Rockville, MD: Agency for Healthcare Research and Quality (AHRQ); 2010.

[6] Ali AK (2011). Pharmacovigilance analysis of adverse event reports for Aliskiren Hemifumarate, a first-in-class direct renin inhibitor. Therap Clin Risk Manag.

[7] (2012). FDA Adverse Event Reporting System (FAERS). US Food and Drug Administration (FDA).

[8] (2012). Code of Federal Regulations Title 21. US Food and Drug Administration (FDA).

[9] DuMouchel W (1999). Bayesian data Mining in large Frequency Tables, with an Application to the FDA Spontaneous Reporting System (with Discussion). Am Statistician.

[10] Bate A, Edwards IR, Hartzema AG, Tilson HH, Chan KA (2008). Data Mining Techniques in Pharmacovigilance. Pharmacoepidemiology and Therapeutic Risk Management.

[11] (April 14, 2011). Drug Safety Communication. Safety Review Update on Reports of Hepatosplenic T-cell Lymphoma in Adolescents and Young Adults Receiving Tumor Necrosis Factor (TNF) Blockers, Azathioprine and/or Mercaptopurine. Food and Drug Administration (FDA).

[12] Fishman JA (2013). Overview: cytomegalovirus and the herpesviruses in transplantation. Am J Transplant.

[13] Piselli P, Busnach G, Fratino L (2013). De novo malignancies after organ transplantation: focus on viral infections. Curr Mol Med.

[14] Gutierrez-Dalmau A, Campistol JM (2007). Immunosuppressive therapy and malignancy in organ transplant recipients: a systematic review. Drugs.

[15] Hengster P, Pescovitz MD, Hyatt D, Margreiter R (1999). Cytomegalovirus infections after treatment with daclizumab, an anti IL-2 receptor antibody, for prevention of renal allograft rejection. Transplantation.

[16] Bechstein WO (2000). Neurotoxicity of calcineurin inhibitors: impact and clinical management. Transplant Int.

[17] Bielory L, Gascon P, Lawley TJ (1986). Serum sickness and haematopoietic recovery with antithymocyte globulin in bone marrow failure patients. Br J Haematol.

[18] Mahmud n, Klipa D, Ahsan N (2010). Antibody immunosuppressive therapy in solid-organ transplant: part I. MABS.

